# Carbon footprint in trauma surgery, is there a way to reduce it?

**DOI:** 10.1186/s44158-024-00181-3

**Published:** 2024-07-17

**Authors:** Elize W. Lockhorst, Philip M. J. Schormans, Cornelis A. S. Berende, Pieter Boele van Hensbroek, Dagmar I. Vos

**Affiliations:** 1 Department of Surgery, Amphia Hospital, Molengracht 21, 4818 CK Breda, The Netherlands; 2 Department of Surgical Oncology and Gastrointestinal Surgery, Erasmus MC University Cancer Institute, Dr. Molewaterplein 40, Rotterdam, 3015 GD The Netherlands

**Keywords:** Traumasurgery, Carbon footprint, Anaesthesia

## Abstract

**Background:**

Inhaled anaesthetic agents like sevoflurane contribute for approximately 5% to healthcare’s carbon footprint. Previous studies suggested that the use of these agents should be minimized. Although multiple trauma surgeries can be performed under regional anaesthesia, most are performed under general anaesthesia. This study aims to evaluate the environmental benefits of using regional anaesthesia over general anaesthesia and to compare the associated complication rates.

**Methods:**

This retrospective study included all trauma patients (≥ 18 years) who underwent surgical intervention for hand, wrist, hip, or ankle fractures from 2017 to 2021. The hypothetical environmental gain was calculated based on the assumption that all surgeries were performed under regional anaesthesia. Complication rates were compared between regional and general anaesthesia.

**Results:**

Of the 2,714 surgeries, 15% were hand, 26% wrist, 36% hip, and 23% ankle fractures. General anaesthesia was used in 95%, regional in 5%. Switching this 95% to regional anaesthesia would reduce the sevoflurane use by 92 k, comparable to driving 406,553 km by car. The complication rate was higher with general anaesthesia compared to regional (7.7% vs 6.9%, *p* = 0.75).

**Conclusion:**

The potential gain of the reduction of sevoflurane in trauma surgeries which can be performed under regional anaesthesia can be significant.

## Introduction

The World Health Organization considers climate change the greatest threat to the global human health [1]. A part of this climate change is due to the healthcare system itself; this sector generates a considerable amount of greenhouse gases [2]. The average carbon footprint, from the total percentage of the national footprint, in healthcare is 5.5% (3.3–8.1%) [3]. Campbell et al. described that 5% of the carbon footprint of the National Healthcare Service of the UK is attributable to inhaled anaesthetic agents [4].

Since 1844, anaesthetic gases have been in clinical use [5]. Nowadays, the most used volatile anaesthetics are dinitrogen monoxide (N_2_O), sevoflurane, desflurane, and isoflurane, all of which contribute to climate change by modifying the earth’s atmosphere [6]. N_2_O and gases such as isoflurane deplete the ozone layer, while gases such as sevoflurane and desflurane do not damage the ozone layer. These greenhouse gases are harmful because they cause global warming by absorbing and reducing outgoing infrared thermal energy [6]. Desflurane has the biggest environmental impact among the anaesthetic gases compared to sevoflurane and isoflurane. The combination of N_2_O with sevoflurane or isoflurane significantly increases the impact of global warming, but if N_2_O is combined with desflurane, this impact decreases [7]. However, the destructive impact of N_2_O on the ozone layer does not make it favourable for use [7]. Therefore, previous studies suggested that the use of inhaled anaesthetics should be minimized as much as possible to reduce the carbon footprint of anaesthesia [6, 7]. In order to reach this goal, regional anaesthesia instead of general anaesthesia may be considered. Kuvadia et al. presented a theoretical calculation of the reduction of greenhouse gas emissions when performing hip and knee arthroplasties under regional anaesthesia. This is one of the few studies, or perhaps the only one, presenting the environmental impact of regional versus general anaesthesia of a specific surgery [8]. This paper showed, theoretically, an emission of 112,000 kg of desflurane and 9,000 kg of N_2_O if all 1,124,000 hip and knee arthroplasties performed in the USA in 2009 were done under general anaesthesia. This equals 11.8 million kilometres driven by an average passenger vehicle [8].

In trauma surgery, multiple surgeries can be performed under regional anaesthesia as well, such as surgeries of the hand, wrist, hip, and ankle. In this study, we extracted data regarding the anaesthesia used for these types of surgeries with the aim to determine how often these surgeries were performed under general anaesthesia when they could have been performed under regional anaesthesia, as well as the potential environmental benefits of performing these surgeries under regional anaesthesia. Additionally, we assessed the differences in complications between the use of general and regional anaesthesia.

## Materials and methods

### Study design

The data for this retrospective study were collected in a large teaching hospital in the Netherlands between January 2017 and December 2021. We analyzed the frequency of surgeries performed under general anaesthesia when regional anaesthesia could have been utilized. Furthermore, we investigated the potential environmental advantages of conducting these surgeries under regional anaesthesia. This environmental gain was estimated by calculating how much sevoflurane would have been saved if surgeries were performed under regional anaesthesia. We also evaluated the dissimilarities in complications, including mortality rates, wound infections, haemorrhaging, and compartment syndrome, between the use of general and regional anaesthesia.

Approval for this study was obtained from the Medical Ethics Committee (N2022-0490).

### Study population

If patients underwent a surgical procedure for a fracture of the hand, wrist, hip, or ankle between January 2017 and December 2021, they were included in the study. Excluded were all patients under 18 years of age or if documentation of anaesthesia was unavailable. The patients were divided into two groups: the group who underwent general anaesthesia and the group who underwent regional anaesthesia. The patients in the regional anaesthesia group did not receive any sedation or supplementary analgesia.

### Data collection

Patient data collected from the patients’ records were age, sex, type of fracture, type of anaesthesia, type of surgery, and postoperative complications. Additionally, we collected information on the total number of surgeries and the type of anaesthesia used for all procedures in this hospital. Finally, we gathered data on the total consumption of sevoflurane over these years. We obtained this sevoflurane consumption data from the pharmacy by recording the number of 250 ml vials utilized.

### Data analysis and statistics

The normal distribution of the data was tested. In the case of a normal distribution, the mean and standard deviation were delineated. If data were non-normally distributed, the median and interquartile range (IQR) were reported. χ^2^ tests and Fisher exact tests were utilized to compare categorical variables. All data analyses were performed with IBM SPSS Statistics Program version 25.

In order to calculate the hypothetical environmental gain of minimizing sevoflurane consumption by performing surgeries under regional anaesthesia, the following calculation has been used:

1 $$\text{Percentage of traumasurgeries }= \frac{\text{Fracture}-\text{related surgeries of the hand},\text{ wrist},\text{ hip},\text{ or ankle}}{\text{Total number of surgeries}}$$

2 $$\text{Total amount of sevoflurane }\left(\text{ml}\right)=\text{total number of vials for all surgeries}\times 250$$

3 $$\text{Sevoflurane used for traumasurgeries }\left(\text{ml}\right)=\text{Percentage of traumasurgeries }\times \text{total amount of sevoflurane }(\text{ml})$$

4 $$\text{Sevoflurane used for traumasurgeries }\left(\text{kg}\right)= \frac{(\text{Sevoflurane used for traumasurgeries }\left(\text{ml}\right)\times 1.52)}{1000}$$

5 $$\text{CO}2\text{ emission of traumasurgeries }(\text{kg})=\text{Sevorflurane used for traumasurgeries }\left(\text{kg}\right)\times 440$$

In order to provide a more tangible representation of this amount, the CO_2_ emission was translated into the distance that a car could travel. Similarly, the impact of using sevoflurane for hand, wrist, hip, or ankle and surgeries under general anaesthesia was expressed in terms of car mileage. This calculation was carried out using the following formula:

6 $$\text{Number of kilometres by car}=\frac{\text{CO}2\text{ emisson of traumasurgeries }\left(\text{kg}\right)}{0.1}$$

Further elaboration of the above calculation is as follows: (1) Surgeries performed under general anaesthesia between 2017 and 2021. (2) One vial contains 250 ml of sevoflurane. (4) The density of sevoflurane is 1.52 [9]. (5) One kilogram of sevoflurane is 440 kg of CO_2_ [10]. (6) For every kilometre driven, a car emits 0.1 kg of CO_2_ [10].

## Results

Between January 2017 and December 2021, a total of 117,801 surgeries were performed in our hospital, of which 106,674 were under general anaesthesia.

In this period 2,989 patients underwent a fracture-related surgical procedure of the hand, wrist, hip, or ankle. Based on age, 249 patients were excluded and 26 patients were excluded because no anaesthetic records were available. The remaining 2,714 patients who received surgical treatment were included for analyses. Out of the trauma-related surgeries, 15% involved the hand, 26% involved the wrist, 36% involved the hip, and 23% involved the ankle. The median age of the complete group was 64 years (IQR 47–77), and 61.5% of all patients were female (*n* = 1670).

### General versus regional anaesthesia

Among the total of 2,714 surgeries, a small proportion underwent regional anaesthesia (4.8%). The general anaesthesia group had a median age of 64 years (IQR 47–77), while the regional anaesthesia group had a median age of 72 years (IQR 52–80). Furthermore, the percentage of women was higher in the regional anaesthesia group compared to the general anaesthesia group, with values of 74% and 61%, respectively. Regarding the type of surgery, 22% of patients who underwent general anaesthesia had hand surgery, 37% had wrist surgery, 15% had hip surgery, and 26% had ankle surgery. Conversely, among patients who received regional anaesthesia, 31% underwent hand surgery, 31% wrist surgery, 21% hip surgery, and 17% ankle surgery. Notably, there was a significant difference between general and regional anaesthesia in all categories (Table 1).

**Table 1:** Baseline characteristics

		**General anaesthesia % ** ***n*** ** = 2584**		**Regional anaesthesia % ** ***n*** ** = 130**		***P*** **-value**
*Age (years)*	Median (IQR)	64	(47–77)	72	(52–80)	0.001
*Sex*	Male	1010	(39)	34	(26)	0.003
	Female	1574	(61)	96	(74)	
*ASA* ^*a*^	I	538	(21)	22	(17)	0.608
	II	1030	(40)	59	(45)	
	III	599	(23)	31	(24)	
	IV	92	(4)	7	(5)	
	V	1	(0)	0	(0)	
	Missing	324	(12)	0	(0)	
*Anticoagulants* ^*b*^	No anticoagulants	1696	(66)	88	(68)	0.288
	LMWH^c^	293	(11)	22	(17)	
	PAI^d^	264	(10)	10	(8)	
	VKA^e^	115	(5)	3	(2)	
	P2Y_12_ inhibitors	91	(4)	2	(2)	
	DOAC^f^	77	(3)	6	(5)	
	Missing	193	(8)	4	(3)	
*Body Mass Index (kg/m* ^*2*^ *)*	Mean (min–max)	26.6	(15.6–51)	25.1	(19.2–33.4)	0.24
*Type of fracture*	Hand	573	(22)	41	(32)	0.005
	Wrist	949	(37)	40	(31)	
	Hip	384	(15)	27	(21)	
	Ankle	678	(26)	22	(17)	

^a^The American Society of Anesthesiologists Classification

^b^Some patients had multiple anticoagulants

^c^Low molecular weight heparin

^d^Platelet aggregation inhibitor

^e^Vitamin k antagonist,

^f^Direct oral anticoagulant

### CO_2_ emission of general anaesthesia

**Fig. 1:**
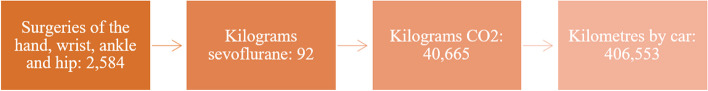
The overall carbon dioxide emissions resulting from general anaesthesia during surgeries on the hand, wrist, hip, and ankle

**Fig. 2:**
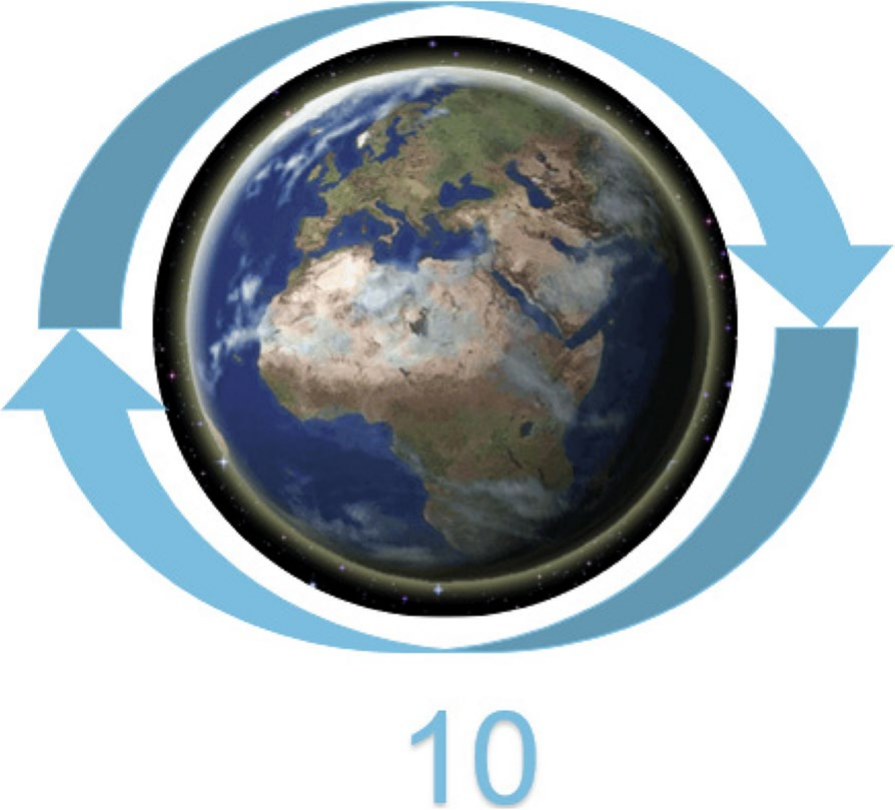
The overall carbon dioxide emissions resulting from general anaesthesia during surgeries on the hand, wrist, hip, and ankle, translated into the number of laps that could be driven around the world

Between 2017 and 2021, out of all surgeries performed under general anaesthesia in this hospital, 2.4% were surgeries of the hand, wrist, hip, or ankle. For all of these surgeries, 10,038 vials of sevoflurane were used, which is equivalent to 243 vials for this 2.4%. This is equal to 60,788 ml of sevoflurane, which is the same as 92.398 g or 92 kg. The total CO_2_ emission from this amount is 40,655 kg of CO_2_. When converted to the number of kilometres you can drive by car for the same emission, this is 406.553 km (Fig. 1). This is comparable to driving ten times around the world (Fig. 2).

### Complications

Of all patients, 208 (7.7%) patients had one or more postoperative complications, with a total of 239 complications. For patients who underwent surgery with general anaesthesia, 7.7% had a complication; for patients who underwent regional anaesthesia, this percentage was 6.9%. However, this difference was not significant.

Complications such as delirium, postoperative bleeding, compartment syndrome, and death did not occur among patients who underwent surgery with regional anaesthesia (Table 2).

**Table 2:** Complications postoperative

		**General ** ***n*** ** = 2584**	**%**	**Regional ** ***n*** ** = 130**	**%﻿**	***P*** **-value**
*Complication*	No	2385	(92)	121	(93)	0.745
	Yes	199	(8)	9	(7)	
*Type of complication*	Delirium	47	(2)	0	(0)	
	Wound infection	42	(2)	2	(2)	
	Bleeding postoperative	18	(1)	0	(0)	
	Compartment syndrome	3	(0)	0	(0)	
	Loosening of breakage osteosynthesis	37	(1)	4	(3)	
	Death	3	(0)	0	(0)	
	Other^1^	79	(3)	4	(3)	

^1^Other complications: acute kidney failure, anaemia, atrial fibrillation, cerebrovascular accident, heart failure, electrolyte insufficiency, fall in hospital, haematoma, haematuria, ileus, infection of osteosynthesis, lung embolism, management problems, melaena, myocardial infarction, necrosis of the wound, osteitis, pneumonia, pressure ulcer, transient ischemic attack, ulnar nerve dysfunction, urinary retention, and urinary tract infection

## Discussion

In this study, we calculated the number of surgeries on the hand, wrist, hip, and ankle, and determined how often these surgeries were performed under general and regional anaesthesia. This was done to theoretically calculate the potential amount of sevoflurane that could be saved if more surgeries were performed under regional anaesthesia. This amount is, just looking at the data of our hospital, considerable. If all of these surgeries were performed under regional anaesthesia, the amount of sevoflurane used could be reduced by 92 kg. Although complications were more frequent with general anaesthesia, the difference was not statistically significant.

Only a few previous studies have been conducted to estimate the CO_2_ emissions associated with specific surgical procedures and the potential benefits of performing these procedures under regional anaesthesia. Kuvadia et al. calculated that all hip and knee arthroplasties performed in the USA in 2009 resulted in a total burden of 112,000 kg of desflurane and 9,000 kg of nitrous oxide, equivalent to a carbon footprint of 7.35 million miles driven by an average passenger vehicle [8]. Another study comparing general and spinal anaesthesia for transforaminal lumbar interbody fusion found that the median CO_2_ emissions associated with general anaesthesia were 4,725 g, whereas those associated with spinal anaesthesia were 70 g (*p* < 0.01) [11]. Both studies suggest a potentially positive impact of performing surgeries under regional anaesthesia on the environment.

Greaves et al. described the concern of the negative environmental impact of regional anaesthesia compared to general anaesthesia due to the increased use of single-use plastics. Although, they compared a surgical procedure performed under general and regional anaesthesia, considering factors like gases, travel, single-use items, other drugs, and anaesthesia-related energy use. The CO_2_-equivalent emissions were lower for regional anaesthesia compared to general anaesthesia (6.4 kg vs 10.7 kg) [12].

However, one other study showed no difference in the carbon footprint between regional and general anaesthesia. Although, the study design of this study was not similar to our study design. In our study, we only focussed on the use of sevoflurane; McGain et al. have conducted a more extensive analysis. Along with sevoflurane, they incorporated all aspects of the surgical process in a life cycle study. However, in their study, only patients who underwent a knee replacement were included. Our study did not include this type of surgery. In addition, the number of included patients between these two studies differed considerably, 30 vs 2,714 patients [13].

In the literature, the use and environmental impact of anaesthetic gases are mainly clarified by expressing the CO_2_ emission and, for example, the number of kilometres driven by car, as we have done. However, Slingo et al. suggest that this may not be the most accurate way to describe the environmental impact. They state that these calculations misdirect efforts away from addressing major sources of CO_2_ and other significant greenhouse gases, which have long-term and substantial climate effects. Converting the use of aesthetic gases directly into CO_2_ emissions or driven kilometres may be misleading because it oversimplifies the complex environmental impacts of these gases. In addition, they state that emission metrics like GWP (global warming potential) and CO_2_e (CO_2_ equivalent) place undue emphasis on volatile aesthetic agents within healthcare carbon footprints [14].

There is no consensus in the literature about the complications of general and regional anaesthesia. In our study, we found a higher complication rate in the patients who underwent general anaesthesia, although this was not significant. A large study by Ahn et al., including 51,186 patients who underwent hip fracture surgery, demonstrated a lower mortality rate (2.24% vs 2.55%, *p* = 0.0047), lower delirium incidence (20.27% vs 22.77%, *p* < 0.001), and lower incidence of intensive care unit stays (22.81% vs 31.47%, *p* < 0.0001) among patients who had regional anaesthesia [15]. Another study including patients with hip fractures showed a higher rate of pulmonary complications in the general anaesthesia group and a higher rate of cardiac complications in the regional anaesthesia group (*p* = 0.017 and *p* = 0.011, respectively) [16]. On the other hand, other studies for these fractures did not find a significant difference in delirium, mortality, and other complications [16, 17]. For fractures of the upper and lower extremity, no significant differences were found in complications, readmissions, and mortality between general and regional anaesthesia [9, 18, 19].

Regional anaesthesia, especially peripheral nerve blockade, can theoretically mask acute compartment syndromes. In our study, only three patients developed compartment syndrome, and all of them had general anaesthesia. A recent systematic review showed that two studies reported a delay in the diagnosis of compartment syndrome, while four studies did not. At this moment, there is no consensus in the literature about the safety of peripheral nerve blockade and the diagnosis of compartment syndrome. Of course, we should always stay alert that compartment syndrome can occur [20].

In addition to complications, other factors could be decisive in choosing a particular type of anaesthesia. Regarding functional outcomes, a number of studies have reported no statistically significant differences at 6-month follow-up [18, 21]. However, patients who received regional anaesthesia did report significantly fewer pain complaints during the first 24 h after the procedure, although this effect diminished thereafter [21, 22]. Consequently, it cannot be definitively asserted that one type of anaesthesia is superior to the other in terms of complications or functional outcomes. Either type of anaesthetic appears to be safe and effective in performing these surgeries. However, not all patients are suitable candidates for regional anaesthesia due to various reasons, such as ongoing infection at the needle insertion site, allergy to local anaesthetics, coagulopathy, and the use of certain anticoagulants [23–25].

When interpreting our results, it is important to consider some limitations. The first and major limitation is that this study only considered the hypothetical reduction in sevoflurane usage if all trauma-related surgeries of the hand, wrist, hip, and ankle would be performed under regional anaesthesia. However, if all of these surgeries would be performed under local anaesthesia, other products may be used that could also contribute to CO_2_ pollution, for example, specific types of medicine, chlorhexidine, cleaning sets, and sterile gloves. As described by Shelton et al., to accurately determine the actual difference in CO_2_ emissions between general and regional anaesthesia, these factors must also be taken into account [26]. On the other side, the use of regional anaesthesia results, for example, in a reduced need for disposable tubes and laryngeal masks. Furthermore, patient, surgeon, and anaesthetist preferences also have a significant influence on the decision-making process, which may lead to the preference for general anaesthesia. The final limitation pertains to the number of patients, where there is a substantial discrepancy in the patient count between the general and regional anaesthesia groups. Consequently, making a valid comparison of complications between the two groups becomes challenging.

## Conclusion

The potential benefits of reducing sevoflurane usage and subsequent CO_2_ emissions in trauma surgeries can be significant, just by performing more surgeries under regional anaesthesia. Therefore, it is important to realize that trauma-related surgeries of the hand, wrist, hip, and ankle that are performed under general anaesthesia contribute in a negative way to climate change. For each type of surgery, also in trauma surgery, it is worth considering if surgery under local anaesthesia is a feasible option, so that we can take our responsibility in this part of the health care system.

## Data Availability

The datasets used and/or analysed during the current study are available from the corresponding author on reasonable request.
